# Molecular microbial ecology of stable versus failing rice straw anaerobic digesters

**DOI:** 10.1111/1751-7915.13438

**Published:** 2019-06-10

**Authors:** Andrew M. Zealand, Ran Mei, Anthony P. Roskilly, WenTso Liu, David W. Graham

**Affiliations:** ^1^ School of Engineering Newcastle University Newcastle upon Tyne NE1 7RU UK; ^2^ Department of Civil and Environmental Engineering University of Illinois at Urbana‐Champaign 205 North Mathews Ave Urbana IL 61801 USA; ^3^ Sir Joseph Swan Centre for Energy Research Newcastle University Newcastle upon Tyne NE1 7RU UK

## Abstract

Waste rice straw (RS) is generated in massive quantities around the world and is often burned, creating greenhouse gas and air quality problems. Anaerobic digestion (AD) may be a better option for RS management, but RS is presumed to be comparatively refractory under anaerobic conditions without pre‐treatment or co‐substrates. However, this presumption assumes frequent reactor feeding regimes but less frequent feeding may be better for RS due to slow hydrolysis rates. Here, we assess how feeding frequency (FF) and organic loading rate (OLR) impacts microbial communities and biogas production in RS AD reactors. Using 16S rDNA amplicon sequencing and bioinformatics, microbial communities from five bench‐scale bioreactors were characterized. At low OLR (1.0 g VS l^−1^ day^−1^), infrequently fed units (once every 21 days) had higher specific biogas yields than more frequent feeding (five in 7 days), although microbial community diversities were statistically similar (*P* > 0.05; ANOVA with Tukey comparison). In contrast, an increase in OLR to 2.0 g VS l^−1^ day^−1^ significantly changed Archaeal and fermenting Eubacterial sub‐communities and the least frequency fed reactors failed. ‘Stable’ reactors were dominated by *Methanobacterium*, *Methanosarcina* and diverse *Bacteroidetes*, whereas ‘failed’ reactors saw shifts towards *Clostridia* and *Christensenellaceae* among fermenters and reduced methanogen abundances. Overall, OLR impacted RS AD microbial communities more than FF. However, combining infrequent feeding and lower OLRs may be better for RS AD because of higher specific yields.

## Introduction

Anaerobic digestion (AD) is a biological treatment process that converts organic carbon into carbon dioxide (CO_2_), methane (CH_4_) and other gases while also reducing organic solids masses (Angelidaki and Sanders, 2004). AD systems use a wide variety of substrates, including household wastes, food, sewage and crop residues, separately or in combination (Lim *et al.*, 2012). One possible substrate is rice straw (RS), which is an agricultural waste containing high levels of potential energy as organic solids and lignocellulose (Mussoline *et al.*, 2013). Huge volumes of RS are generated in global agriculture, but disposed of inappropriately, such as by open burning, which causes massive health and environmental problems, especially in China and India (Zhiqiang *et al.*, 2011).

An alternate option for managing waste RS is AD, which has the added benefit of recovering potential energy as CH_4_. However, as a biological system, RS AD has not been optimized and also is subject to shock changes due to volatile fatty acid (VFA) accumulation or pH decreases, causing system failure producing little or no CH_4_ (Tait *et al.*, 2009; Franke‐Whittle *et al.*, 2014). Further, given that most worldwide RS production is from small‐to‐medium sized rural farms, AD processes must be workable at smaller scales and compatible with the acyclic nature of harvests and RS production. Therefore, issues such as feeding frequency (FF) and organic loading rates (OLR) become critical to making the technology sustainable.

Zealand *et al. *(2017) recently showed that less frequent feeding of RS AD units can generate higher specific CH_4_ yields than more frequently fed units at low OLRs, which is promising. However, higher OLRs caused the most infrequently fed reactors to fail, but how failure and general operating conditions impact resident AD microbial communities has not been assessed. In particular, how RS AD microbial populations respond to different FFs and OLRs is largely unknown, especially for shock loads (Mei *et al.*, 2016b). Some studies have investigated microbial communities in RS AD (Nakakihara *et al.*, 2014; Yan *et al.*, 2015; Chen *et al.*, 2016), but the range of conditions has been limited. Further, no studies have assessed the effect of different FFs on AD microbial community conditions. Addressing this knowledge gap is key because it will help better explain performance stability in RS AD and also system failure, which likely depends on subtle ecological interactions at the microbial scale. Zealand *et al. *(2017) showed VFA and VS accumulation, as a product of infrequent mass loading at extreme feeding frequencies, was found to be key in reactor failure, suggesting syntrophic degradation of such acids is a critical step to stability.

To determine the relative effect of FF and OLR on bacterial and Archaeal communities, we characterized 16S rRNA genes using Illumina MiSeq to assess how microbial community structure and diversity varied in reactors under different physiochemical operating conditions, including five FFs and two OLRs. Identifying predominant microorganisms and possible ecological interactions was a key aim of this study to understand AD system stability and also identify potential biomarkers to guide biostimulation interventions.

## Results and discussion

### Impact of FF and OLR on β‐diversity and physiochemical parameters

**Figure 1:**
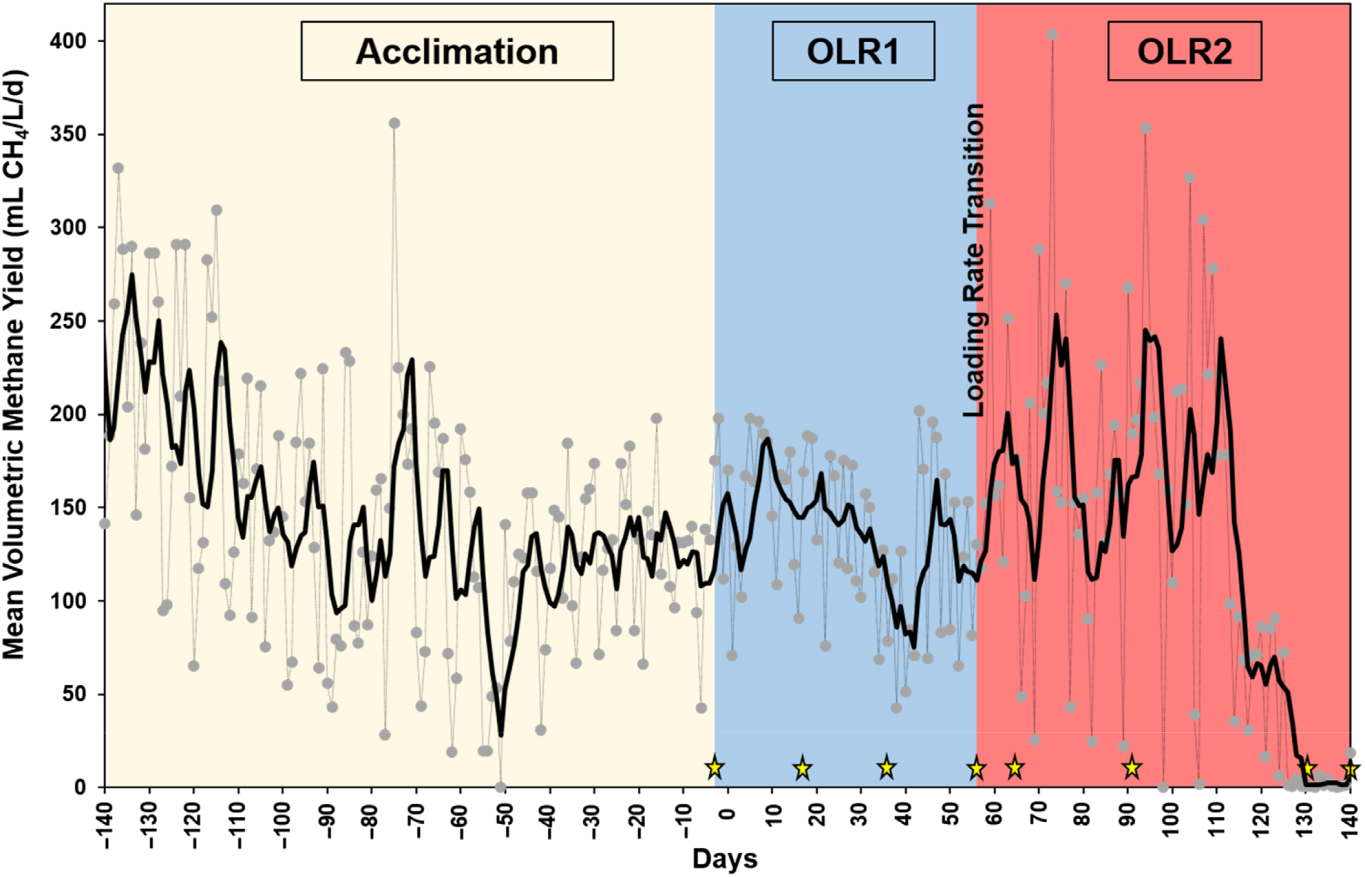
Time‐course volumetric mean methane yield data for the reactor operated with one feed per 14 days (1/14) as a representative example. Showing, stabilization phase (day −140 to −50), fully stable (day −50 to 0), loading rate at 1 g VS l^−1^ day^−1^ (OLR1) from day 0 to 56 and loading rate at 2 g VS l^−1^ day^−1^ (OLR2) from day 56 to 140. Grey points and line show individual data points while black line shows rolling average trends. Star symbols indicate days at which microbial samples were collected (i.e. days 0, 17, 36, 56, 64, 92, 132 and 140).

The experimental timeline related to microbial community analyses is summarized Fig. 1. Highest specific CH_4_ yields were observed in the reactor fed once every 21 days (1/21; FF) at the lower OLR (OLR1; 148 ml CH_4_^ ^g VS^−1^ day^−1^), whereas the highest volumetric yields were seen in the 1/7 reactor (once every 7 days) at the higher OLR (OLR2; 276 ml CH_4_ l^−1^ day^−1^) (Zealand *et al.*, 2017). At OLR2, both the 1/14 (once every 14 days) and 1/21 (once every 21 days) FF reactors failed, apparently due to volatile fatty acid (VFA) accumulation and evident reactor acidification. Both conditions reached mean total VFA levels of 1730 and 3470 ppm respectively (see Table 1; Zealand *et al., *(2017)). As seen in the 1/7 reactor at OLR2, recovery from such acid levels is possible (also Kawai *et al.*, 2014), but also can be irreversible and cause biomethane yield failure (Neves *et al.*, 2004). In contrast, at OLR2, the 5/7 (five in 7 days) and 3/7 (three in 7 days) reactors remained stable throughout as evidenced by consistent methane production and physiochemical conditions.

**Table 1:** Overall mean performance data for reactors with different feeding regimes and organic loading rates as reproduced from Zealand *et al. *(2017).

Feed frequency	5/7^a^		3/7		1/7		1/14		1/21	
Organic loading rate (g VS l^−1^ day^−1^)	1.0	2.0	1.0	2.0	1.0	2.0	1.0	2.0	1.0	2.0
Biogas (ml g VS^−1^ day^−1^)	301 ± 8.4^b^	239 ± 5.1	299 ± 6.8	215 ± 4.6	**317** ±** 8.8** c	249 ± 5.8	295 ± 9.9	139 ± 10.0	303 ± 11.5	42.0 ± 7.8
% CH_4_	40.2 ± 1.3	52.1 ± 1.4	42.4 ± 1.2	52.2 ± 1.7	46.7 ± 1.7	**55.4 ± 1.7**	45.4 ± 1.4	38.7 ± 2.4	49.3 ± 1.4	21.7 ± 1.4
Specific CH_4_ (ml CH_4_ g VS day ^−1^)	112 ± 4.6	125 ± 4.4	127 ± 4.5	112 ± 4.4	146 ± 6.0	138 ± 5.3	134 ± 6.0	63.4 ± 5.6^d^	**148 ± 6.3**	7.7 ± 0.7^d^
Volumetric CH_4_ (ml CH_4_ l^−1^ day^−1^)	112 ± 4.6	251 ± 8.7	127 ± 4.5	224 ± 8.7	146 ± 6.0	**276 ± 10.6**	134 ± 6.0	127 ± 11.1	148 ± 6.3	15.4 ± 1.3
g VS l^−1^	25.9 ± 0.5	38.3 ± 1.7	25.7 ± 0.6	37.0 ± 1.8	25.4 ± 0.8	41.1 ± 2.0	26.9 ± 1.0	43.8 ± 2.6	27.1 ± 1.2	54.1 ± 3.4
% VS Reduction	**44.1 ± 1.8**	41.6 ± 2.3	42.5 ± 1.9	42.8 ± 1.9	42.5 ± 1.8	40.5 ± 2.2	39.4 ± 2.6	31.7 ± 1.6	38.0 ± 3.2	41.3 ± 3.0
Total VFA (ppm)	147 ± 29.4	432 ± 109	135 ± 18.2	495 ± 163	252 ± 43.7	383 ± 57.8	354 ± 77.2	1730 ± 336	1250 ± 312	3470 ± 355
pH	6.8 ± 0.02	6.7 ± 0.01	6.8 ± 0.02	6.7 ± 0.01	6.8 ± 0.01	6.7 ± 0.01	6.7 ± 0.01	6.3 ± 0.06	6.6 ± 0.02	5.7 ± 0.04

a The feeding frequency of each reactor, for example 5/7 = fed 5 days out of seven, 1/21 = fed one day out of twenty one. All feeding frequencies have the same net loading of 1g VS l^−1^ day^−1^ then 2g VS l^−1^ day^−1^.

b Standard error (For OLR 1.0 g VS l^−1^ day^−1^ *n* = 56 for biogas and methane, *n* = 9 for VS; *n* = 13 for VFA and, *n* = 30 for pH; For OLR = 2.0g VS l^−1^ days^−1^ *n* = 84 for biogas and methane, *n* = 12 for VS; *n* = 21 for VFA and, *n* = 44 for pH).

c Bold indicates the highest performing condition for biogas, %CH_4_, specific and volumetric methane yields.

d These reactors failed at the higher OLR of 2 gVS l^−1 ^day^−1^. 1/14 day failed approximately halfway through the experiment and 1/21 days failed immediately.

**Figure 2:**
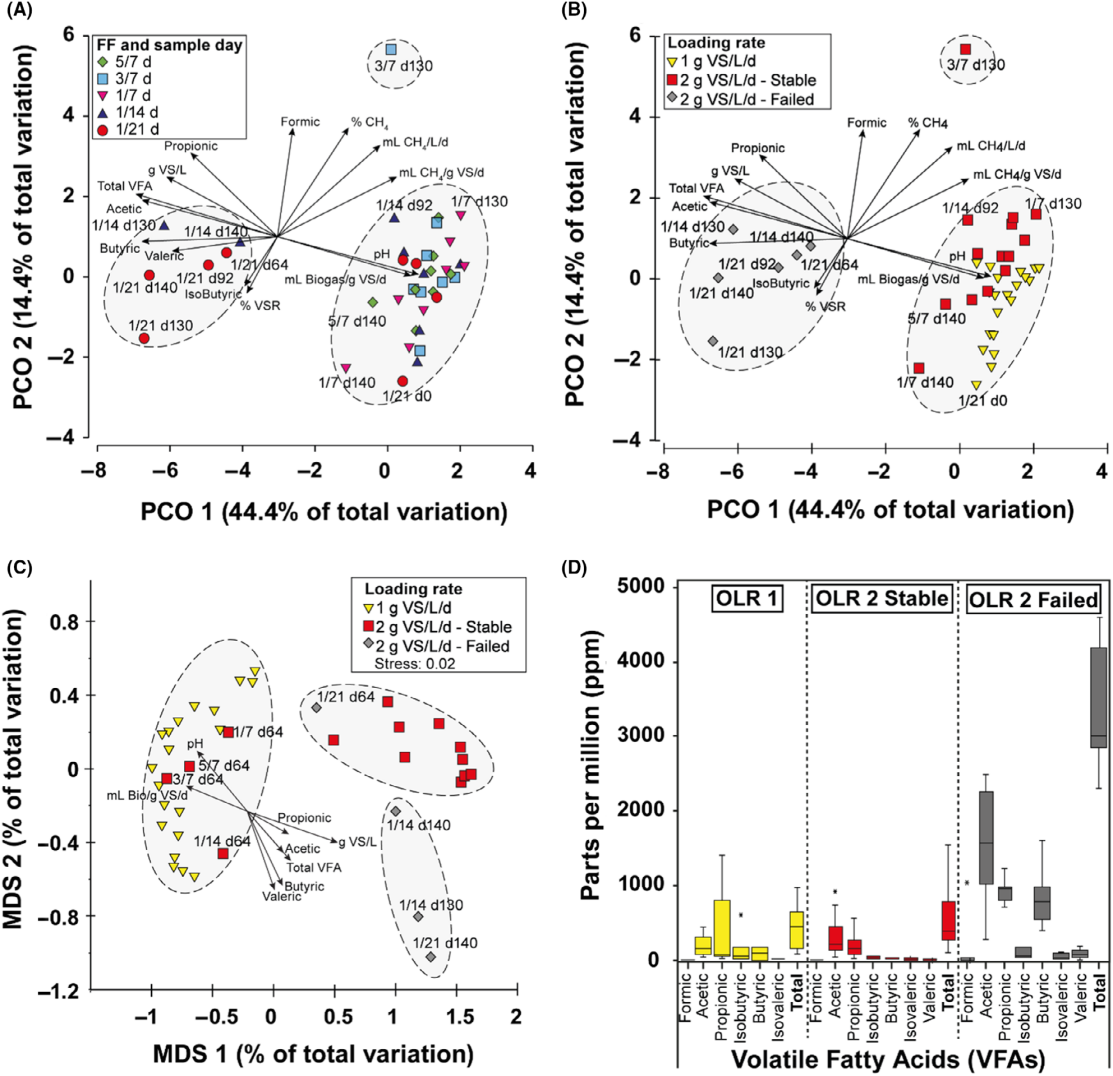
Analyses of β‐diversity showing variation of microbial community structure and the influence of physiochemical data. A and B. Are same figures based on PCA of Bray–Curtis distance, but coloured differently by FF or OLR. C. MDS of weighted UniFrac distance and has two 1/21 days samples fewer than A/B. D. boxplot of individual and total VFAs for OLR1, OLR2‐S and OLR2‐F (there was no valeric acid detected in OLR1). Physiochemical data overlaid arrows and dashed elliptical shapes indicate sample groupings).

Comparing data from all reactors, clustering analysis based on Bray–Curtis distance indicated samples did not cluster based on FF (Fig. 2A) or OLR (Fig. 2B), but primarily grouped into ‘stable’ versus ‘failed’ reactor clusters (3/7 d130 is an outlier). In Fig. 2B, OLR1 samples were all ‘stable’, whereas OLR2 data split between ‘stable’ and ‘failed’ (see below), which are subsequently designated as ‘OLR2‐S’ and ‘OLR2‐F’ respectively. The influence of physiochemical conditions on microbial community is shown by direction and length of corresponding arrows. VFA and VS build‐up related to OLR2‐F samples, whereas all other OLR1 and OLR2‐S samples associated with higher biogas production and pH values. This infers physiochemical differences between reactor conditions were strongest when reactors failed due to OLR rather than FF.

Further clustering analysis based on weighted UniFrac distances (Fig. 2C) grouped OLR conditions into three subsets. OLR1 samples relate to pH and biogas production, OLR2‐F reactors correlate with VFA and VS, and OLR2‐S reactors fell somewhere between. Higher levels of individual and total VFAs for OLR1, OLR2‐S and OLR2‐F (see Fig. 2D) support observations in Fig. 1A–C that suggest increasing VFAs and decreasing pH with reactor failure at OLR2. In addition, as a transitional stage (from OLR1 to OLR2), day‐64 samples did not uniformly cluster with OLR1 or OLR2; that is, the OLR2‐S day‐64 samples grouped with OLR1, whereas 1/21 day‐64 samples grouped with OLR2‐S samples. In shifting reactor conditions from OLR1 to OLR2, there were a number of staged changes in the microbial community; that is, from OLR1 came ‘transition’ (OLR2‐T: day 64 for all FF), ‘stable’ (OLR2‐S, days 92–130 for 5/7, 3/7 and 1/7, as well as day 92 at 1/14) and, ‘failed’ (OLR2‐F, day 130 and 140 for 1/14 with day 140 of 1/21).

To statistically examine the effects of FF and OLR on RS AD β‐diversity and also identify any statistical correlations with the physiochemical data, analyses including RELATE, BEST, DistLM, ANOSIM and PERMANOVA were performed and are summarized in Table 2. RELATE and PERMANOVA provide ‘*P*‐values’ as percentages, that is, 0.1% is equivalent to *P* = 0.001. The influence of physiochemical data was best reflected by a significant (0.1%) 0.40 correlation with β‐diversity (RELATE). BEST and DistLM showed VS and pH followed by butyric acid most correlated with most community structure (BEST, *R* = 0.77 correlation; DistLM, *P* = 0.001). ANOSIM and PERMANOVA analysis confirm the impact of changing FF was not significant, whereas OLR was significant at 0.02% (*P* = 0.001).

**Table 2:** Test statistics for observed β‐diversities, physiochemical variables and other operational factors.

Method: Relate^a^	Significance (%)	Rho.
Variable		
Physiochemical data	**0.1** b	0.403

Method: Best	Physiochemical Correlation (R)
Variable	
g VS l^−1^	0.772
+ pH	0.768

Method: DistLM	*P*‐value	Cumulative variance explained (%)
Variable		
g VS l^−1^	**0.001**	63.9
+ pH	**0.001**	72.7
+ Butyric acid	**0.001**	73.8

Method: ANOSIM	Global R	Significance level (%)
Factor		
Feeding frequency	−0.055	75.5
Organic loading rate	0.532	**0.02**

Method: PERMANOVA	*P*‐value	Square root of estimates of component of variation
Factor		
Feeding frequency	0.823	−2.08
Organic loading rate	**0.001**	11.0
FF × OLR	0.959	−3.58

a Tests – RELATE, giving correlation of comparisons (Rho); ANOSIM, analysis of similarities; BEST, trend correlation; DistLM, distance‐based linear model; PERMANOVA, permutational multivariate analysis of variance.

b Bold indicates statistically significant results.

### Impact of OLR and reactor failure on α‐diversity

**Figure 3:**
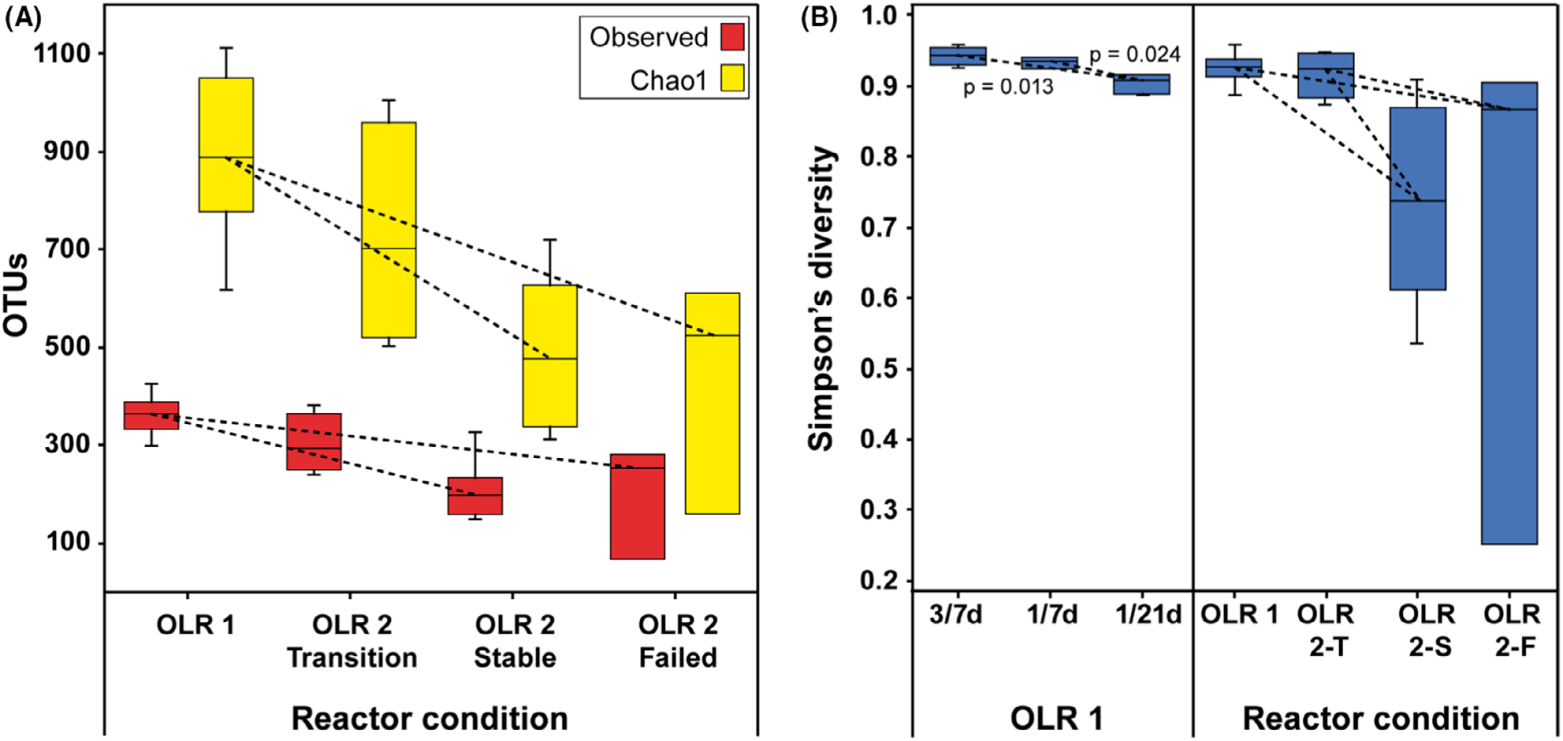
Boxplots between, (A) Observed OTUs (lower) and Chao1 (upper boxes) and (B) Simpson’s index scores at OLR1 FFs that showed significant differences (left) and the mean scores of OLR1, OLR2‐S and, OLR2‐F. Dashed lines and *P*‐value indicate 2‐sample *t*‐test statistical significance between linked samples.

A comparison of α‐diversity indices was undertaken to identify apparent differences in community richness and evenness vs. operating conditions, and no differences were found among FFs within each OLR (consistent with Colwell and Coddington, 1994; Hughes *et al.*, 2001; Lemos *et al.*, 2011). Chao1 and Simpson’s indices were chosen to compare both individually occurring OTUs and more relatively dominant factions of the microbial community. Significant differences were seen between observed in OTU number and Chao1 estimations for OLR1 and those of OLR2‐S and OLR2‐F (*P* < 0.001). This was not the case between OLR1 and OLR2‐T (*P* = 0.635 and *P* = 0.323 respectively), indicating OLR2‐S and OLR2‐F had lower richness relative to OLR1 (Fig. 3A). These Chao1 data imply differences in observed OTUs outweigh the number of single OTUs among conditions.

A Simpson’s index score was calculated and shown in Fig. 3B to compare relative levels of evenness. OLR1 had greater diversity than OLR2‐S and OLR2‐F (*P* < 0.001), as did OLR2‐T (*P* < 0.001). Within OLR1, there also were differences with 1/21 FF, which was significantly less diverse than the 3/7 (*P* = 0.013) or 1/7 days (*P* = 0.024) units. It had been hypothesized that lower FF reactors might have decreased diversity because infrequent loading might tend to select for K‐strategists (e.g. microbial scavengers). As an example, there may be species within *Methanosarcina*, which might be capable of withstanding environments more hostile to others, including *Methanosaeta* (Conklin *et al.*, 2006). It has been shown that *Methanosarcina* can dominate Archaea associated with frequent feeding and when hydrogenotrophic methanogenesis is required (Conklin *et al.*, 2006).

It is also apparent that OLR2‐S and OLR2‐F had reduced OTU richness and evenness, probably resulting from higher organic loading, VFA levels, lower pH and comparatively poor reactor performance. Wittebolle *et al. *(2009) suggested richness and evenness are essential for ‘happy’ AD systems with increased evenness providing a greater substitutional buffer under high stress situations, such as an increase in OLR. It is possible with more time, all reactors at 2.0 g VS l^−1^ day^−1^ might fail as VFAs may have continued to accumulate. In this case, traditional markers of reactor stability, such as pH, responded more slowly than changes in the microbial community, and the effect of FF and changes in community richness and unevenness might play even more significant roles. Rapid population shifts, such as increases in fermenters, decreases in methanogens or related ratios, could therefore be used as early warnings of forthcoming system instability.

### Predominant OTUs correlated to different stages of the AD

**Figure 4:**
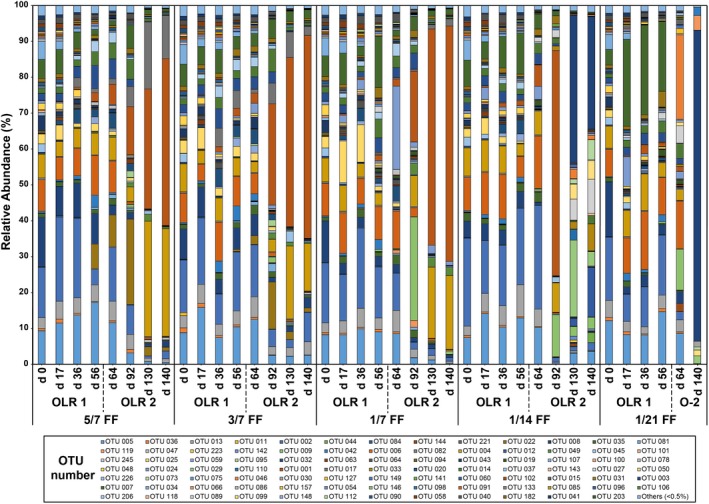
Shared predominant OTU table to genus level (only ≥ 0.5% relative abundance) organized by reactor feeding frequency, loading condition and sampling day.

**Figure 5:**
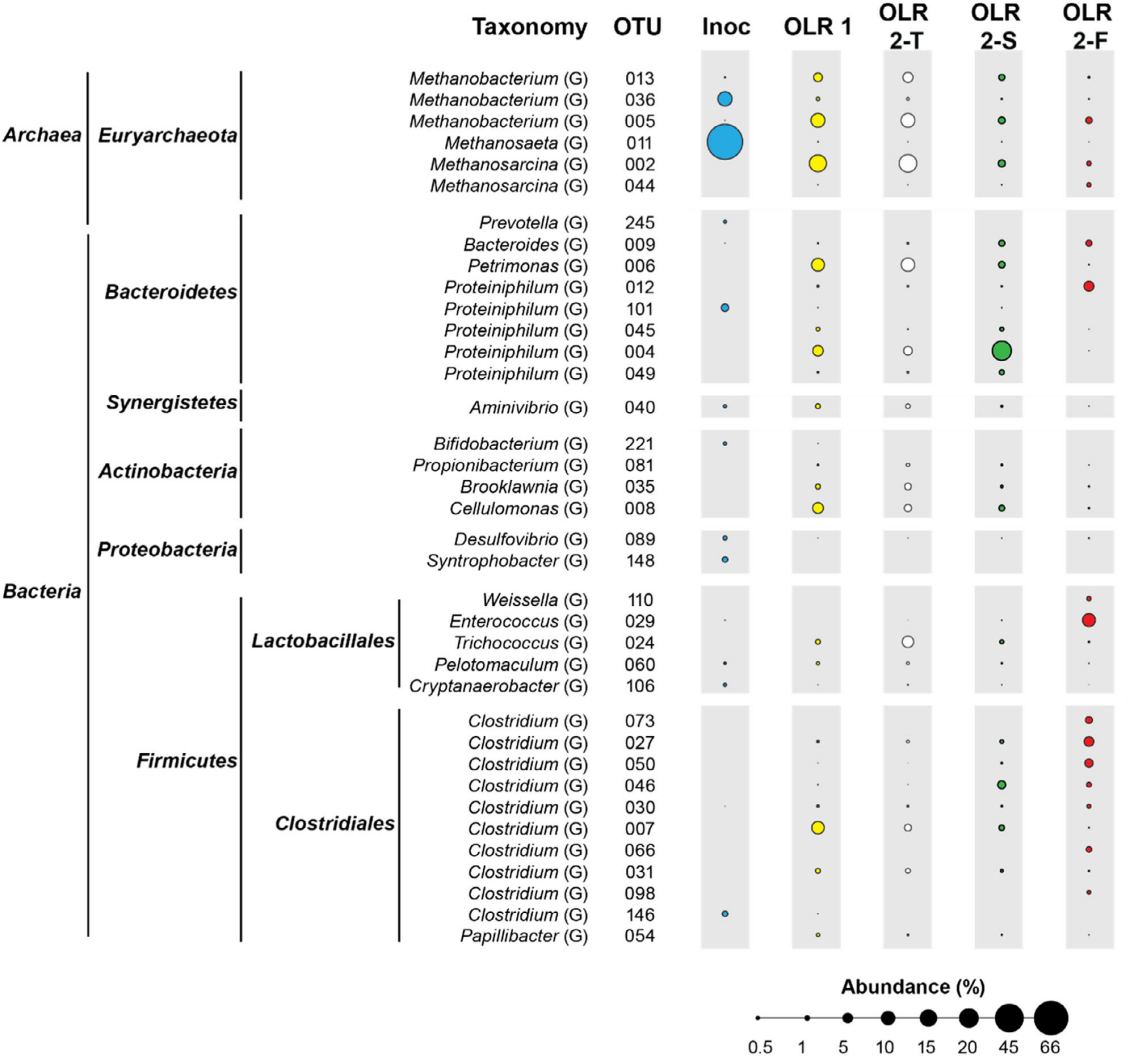
Predominant OTUs (≥ 0.5% abundance) grouped based on ARB phylogenetic tree construction to genus for OLR1, OLR2‐T, OLR2‐S and OLR2‐F. Area of bubbles represents relative abundance. ‘Inoc’ = inoculum. Letters in brackets under ‘Taxonomy’ equate to classification, that is, ‘O’ = Order, ‘F’ = Family and ‘G’ = Genus.

**Figure 6:**
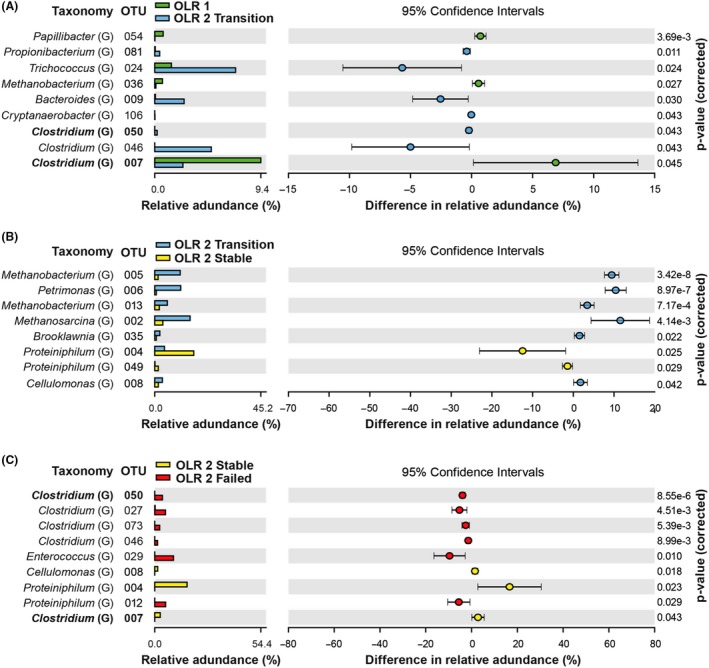
Extended error bar plot showing predominant OTUs to genus level that have significantly different abundances between organic loading conditions, (A) OLR1 and OLR2‐T, (B) OLR2‐T and OLR2‐S and (C) OLR2‐S and OLR2‐F. Only OTUs with ≥ 0.5% abundance are shown, and bold type indicates OTUs that appear in more than one panel.

Changes of predominant OTUs (60 OTUs with a relative abundance ≥ 0.5%) observed under different operating conditions are summarized in Figs 4, 5 and 6. Detailed information related to the phylogenetic affiliation of the 60 OTUs is provided in Figs S2, S3, S4 and S5. Identifying predominant OTUs shared between loading conditions is shown as Fig. S6, which allows us to identify OTUs that are apparently more important to reactor function and allow speculation on how they may interact (similar to Rui *et al.*, 2015; Ling *et al.*, 2016); St‐Pierre and Wright, 2014; Mei *et al.*, 2016a). For example, OTU 002 (*Methanosarcina*), 005 (*Methanobacterium*), 014 (*Christensenellaceae*) and 022 (*Bogoriellaceae*) were found in all samples, and thus were likely to play core roles in the RS AD process as compared with other OTUs that were only found in healthy reactors.

All OLR1 samples were similar and mostly consisted of *Bacteroidetes* and *Firmicutes,* previously noted in straw digestion by Heeg *et al. *(2014)*,* then *Actinobacteria, and Euryarchaeota.* Day 64 (transition, ‘OLR2‐T’) had a similar composition, whereas *Firmicutes* abundance increased dramatically through OLR2. Overall, the number of predominant OTUs in OLR1 (30) declined slightly into OLR2‐T to 25 OTUs. However, the largest drop was from to the OLR2‐S and OLR2‐F operating conditions; 17 and 18 OTUs, respectively, with broad decreases in *Bacteroidetes* and *Actinobacteria* in concert with increased *Firmicutes*. The fact that the number of predominant OTUs in OLR2‐S and OLR2‐F was lower than OLR1 and OLR2‐T implies that differences in OTU presence and abundance in OLR2‐S and OLR‐F potentially relate to OTUs important to optimal reactor performance. Relative abundances of individual OTUs in OLR2‐S and OLR2‐F increased to 44% and 52%, respectively, while the number of OTUs almost halved.

Although overall OTU presence and relative abundance decreased under some AD conditions, methanogens always were present. *Methanobacterium* (OTU 013 and 005) remained steady through OLR1 (4.0% and 10.4%) and OLR2‐T (5.2 and 10.3%) before significantly reducing in OLR2‐S (1.8% and 2.2% at *P* = 0.0007) and OLR2‐F (0.1% and 1.1%). *Methanosarcina* (OTU 002) followed the same general pattern, decreasing over time from 15.2% and 16.6% to 2.6% (*P* = 0.004) and 1.0% respectively. *Methanobacterium* and *Methanosarcina* are among various acidophilic methanogenic Archaea (Bräuer *et al.*, 2006) and are detected through the entire operation, suggesting their probable role in converting hydrogen and acetate to CH_4_ under this low‐pH environment. As indicated previously, *Methanosarcina* appears to be capable of withstanding some environments that can be more hostile to others (Conklin *et al.*, 2006); however, although able to survive at pH lower than 5.0, the optimal pH for *Methanobacterium* is around 6.0 and that for *Methanosarcina* is near neutrality. This difference might explain their declining abundance in OLR2‐F with pH lower than 6.0.

No well‐known obligate syntrophic bacteria, such as *Syntrophobacter*, were observed in high abundance in the reactors, although they were present in the inoculum. This absence is not surprising because it has been observed that obligate syntrophs are often not present in AD systems for agricultural solid waste (Ziganshin *et al.*, 2011; Wang *et al.*, 2012; Sun *et al.*, 2015). This contrasts with microbial communities associated with municipal sludge AD (Mei *et al.*, 2017). The metabolism of syntrophs rely on low hydrogen pressures due to strict thermodynamic requirements (Stams and Plugge, 2009), which may not be the case in RS AD units due to elevated hydrogen production by fermenters. Further studies are needed to elucidate the potential distribution and functions of obligate syntrophs in AD of RS and other high‐energy, but more refractory agricultural substrates.

There was a range of community changes over the course of the experiment*.* Within *Bacteroidetes*, *Petrimonas* (OTU 006) abundance decreased significantly from OLR2‐T (9.5%) to OLR2‐S (2.2%, *P* = 0.03) and then to OLR2‐F (0.0%, *P* = 0.00). While, OTU 004 and OTU 049, associated with *Proteiniphilum* increased significantly from OLR2‐T to OLR2‐S, 3.9–19.6% (*P* = 0.02) to 0.2–1.2% (*P* = 0.03), respectively, before rapidly decreasing in OLR2‐F (0.0%). These fermenters almost wholly produce acetate and H_2_. In contrast, *Petrimonas* is not known to use cellulose (Grabowski *et al.*, 2005) and *Proteiniphilum* do not utilize cellobiose or cellulose (Chen and Dong, 2005). Therefore, it is logical they might decline with increases in a highly cellulosic substrate, such as RS.

Within the phylum *Firmicutes*, *Cellulomonas* (OTU 008) increased during acclimation to OLR1 (to 6.1%), significantly decreased to 2.8% (*P* = 0.042) at the increased loading at OLR2, stabilized at 1.7% in OLR2‐S and declined to 0.1% in OLR2‐F (*P* = 0.018). The decline of *Cellulomonas* was unexpected given the large number of cellulolytic and ligninolytic species within this genus, often associated with the biodegradation of rice plants (Akasaka *et al.*, 2003; Ventorino *et al.*, 2015). Further, there were 10 OTUs associated with *Clostridium* (eight OTUs that increased in relative abundance are discussed below). Among them, OTU 007 and 031 decreased significantly from OLR1 to OLR2‐T (8.4–2.6%, *P* = 0.045) before dropping again into OLR2‐F (*P* = 0.043). OTU 031 was closely matched to a *Clostridium clariflavum* isolate (CP003065.1), which possess a large number of cellulolytic and hemicellulytic enzymes potentially associated with cellulose conversion (Artzi *et al.*, 2015).

There also were some OTUs that only appeared in OLR2‐F reactors. Contrary to declines discussed earlier, *Proteiniphilum* (OTU 012) and *Christensenellaceae* (OTU 033) were both higher in OLR2‐S than OLR2‐F; that is, 0.1% to 5.1% and 3.1% respectively (*P* = 0.029 and 0.018). *Proteteiniphilum* are fermenting bacteria capable of producing acetic and propionic acid, which fits with the increase in acids in this latter condition (Whitman *et al.*, 2015). *Clostridium* (OTU 050, 073, and, 027) increased significantly from OLR2‐S (0.0%, 0.7% and, 0.1%) into OLR2‐F (2.5%, 4.9% and 3.6% at *P* ≤ 0.005), while OTUs 030, 098, 031 and 066 showed small increases of ≤ 1.6%. The *Clostridiales* order, which includes *Christensenellaceae,* has been previously shown to degrade cellulose (Fontes and Gilbert, 2010; Zverlov *et al.*, 2010; and Ziganshina *et al.*, 2015), which explains their increase in conjunction with RS OLR. The anaerobic cellulolytic ability of the microbial community is generally through *Clostridia*, although it contains few species that can directly degrade cellulose. However, it has been found that some *Clostridium* species (e.g. *Clostridium clariflavum*, *C. thermosuccinogenes* and *Clostridium thermocellum*) can use cellulose and/or cellbiose to produce acetate and formate (Li *et al.*, 2011; Lebuhn *et al.*, 2014; Lü *et al.*, 2014).

The facultative anaerobes *Coriobacteriaceae* (family) and *Weissella* (genus) both increased in OLR2‐F reactors (from 0 to 0.6% and 1%), as did *Ruminococcaceae* (family) and *Enterococcus* (genus), which significantly increased from 0 to 52% and 8.9%. *Ruminococcaceae* are known for breaking down carbohydrates in the intestinal tract, but this family also contains a large number of acetogenic species that degrade cellulosic products, such as *Acetivibrio cellulolyticus* (Dassa *et al.*, 2012). The genus *Ruminococcus* has previously been noted to hydrolyze cellulose in the rumen (Sun *et al.*, 2015), while the species *Ruminococcus flavefaciens* and* Ruminococcus champanellensis* can utilize cellobiose as well as cellulose (Sun *et al.*, 2013), which could explain the huge increase in the OLR2‐F samples. In these samples, where high OLR was coupled with reactor failure, there was likely to have been the greatest level of substrate available for this family. *Enterococcus* has a number of species that could thrive in a high load, high cellulose environment (Valdez‐Vazquez *et al.*, 2015). *Enterococcus faecium* was found to increase fermentation rate and acid production from lignocellulosic substrate (Pang *et al.*, 2014) while *Enterococcus saccharolyticus* was found during silage fermentation that degrade cellobiose and decrease pH (Kuikui *et al.*, 2014), and, *Enterococcus saccharolyticus* and *Enterococcus gallinarum* were found producing H_2 _in a microbial consortium composting cellobiose (Adav *et al.*, 2009).

Predominant OTUs shared among loading conditions are shown as a network in Fig. S6, which allows us to identify OTUs that tend to associate with healthy versus failing reactors (similar to Rui *et al.*, 2015; Ling *et al.*, 2016; St‐Pierre and Wright, 2014; and Mei *et al.*, 2016a). In general, many more phyla were shared among healthy reactors (number of common OTUs shown in brackets); that is, *Firmicutes* (six), *Euryarchaeaota* (five), *Bacteroidetes* (four), *Actinobacteria* (two) and *Synergistetes* (one) than were shared with OLR2‐F (mostly *Firmicutes* (three), with two *Euryarchaeaota* (two) and with only one *Bacteroidetes*). Increasing *Firmicutes* and decreasing *Bacteroidetes, Actinobacteria* and *Synergistetes* in the OLR2‐F samples suggest that, although these can be cellulose‐utilizing bacteria, *Firmicutes* may outcompete and/or can cope with more extreme conditions better. Nelson *et al.* (2011) observed a similar core community in ‘healthy’ systems. Further, Ling *et al. *(2016) showed that, although there were relatively few core OTUs, they contained many of the high abundance OTUs; for example, OTU 001 (43.7%), which is also seen here.

## Conclusions

Microbial community conditions and predominant OTUs were not significantly affected by FF at the lower OLR and were generally slow to change with increased OLR. However, an increase in fermentation acidic products at OLR2 in the two highest FF reactors, resulted in reactor failure. Associated with these observations, clear community differences were apparent between low and high OLR as well as between transitional and failed reactors. When OLR was doubled, microbial community activity shifted towards greater reliance on fermenters, such as *Clostridia* and *Christensenellaceae*. This community shift also was seen in reactors that transitioned from stable reactors to reactor failure, such as changes in key methanogens like *Methanosarcina* that decreased in abundance.

As 16S taxonomic assignments are not based on full sequences, they should be interpreted carefully, although results here provide a valuable window to guide further, deeper sequencing analysis. The data also suggest that specific biostimulation, such as adding populations shared by the healthy reactors, might process higher levels of acid as long as ‘selecting’ physiochemical habitat conditions are maintained. Alternately, optimizing operating procedures to better control acid production could provide additional benefit to RS AD.

Overall, data show that OLR rather than FF more strongly impacts microbial community composition and ecology at lower OLRs in RS AD reactors. However, the work has wider implications to any AD unit digesting less degradable substrates. Specifically, we show that less frequent feeding does not impact the core microbial community as long as OLR is moderately low, which explains why high specific CH_4_ yields are observed in low FF units; that is, the substrate (in this case RS) dictates the community, not the feeding regime. The idea of intentionally feeding a biological treatment unit less frequently goes against traditional views, but if the substrate is less degradable, infrequent feeding can improve performance. This effect is analogous to feeding a patient a slow‐release drug where the release rate is designed to match biological need for the drug. Therefore, analogously, feeding RS AD units (or any less degradable substrate) might benefit from infrequent feeding. This now needs to be examined with other ‘less degradable’ substrates to verify that lower FF may be a better strategy for any such AD application.

## Experimental procedures

### Experiment background

Samples were collected from five 2.5 l continuously stirred tank reactors (CSTRs), which were operated for over 250 days at 37°C. Briefly, the reactors were acclimated to RS for 112 days, after which the reactors were considered stable, based on steady pH, VFA and biogas production (analogously ‘day 0’) (see Zealand *et al.*, 2017 for a more complete discussion). The reactors were then operated for 56 days at an OLR rate of 1.0 g VS l^−1^ day^−1^ (OLR1), and then transitioned to an OLR of 2.0 g VS l^−1^ day^−1^ (OLR2), which was operated for 84 days. Throughout, reactors operated at different FFs, including five times in 7 days (5/7), three in seven (3/7), one in seven (1/7), one in 14 (1/14) and one in 21 (1/21). As an example, the 5/7 reactor at OLR1 had 56 ml of reactor volume removed per feed (five times per week) before receiving 2.8 g VS of RS mixed in 56 ml of distilled water. As contrast, the 1/21 reactor had 840 ml volume removed every three weeks before which 47.7 g VS RS and 840 ml distilled water were added. Physiochemical analysis of this first experiment is summarized in Table 1, which aligns with Fig. 1 that provide a graphical representation of experiment transitions (Zealand *et al.*, 2017).

### Sample collection and preparation

The original reactor inoculum and four independent samples per reactor were collected for each OLR from the five reactors (days 0, 17, 36 and 56 for OLR1 and days 64, 92 130 and 140 for OLR2), resulting in 39 total samples. Due to reactor failure, only days 64 and 140 were collected at OLR2 for the 1/21 day reactor. ‘Stable’ reactors were those that were consistent with time in terms of pH and biogas yields, whereas reactors were considered ‘failed’ when biogas yield became almost nil and there was clear acid and VS accumulation. All samples were collected in triplicate and stored at −20°C before further analysis. For each sample, genomic DNA was extracted following the instructions of the FastDNA SPIN Kit for Soil (MP Biomedicals, Carlsbad, CA, USA). Quantification of extracted DNA was performed using a Qubit 2.0 Fluorometer (Invitrogen, Waltham, MA, USA) to ensure quantity for analysis.

### Sequencing analysis

Analysis of the extracted DNA (concentration between 1 and 10 ng µL^−1^ and a volume of 10‐20 µL) was undertaken by LGC Genomics GmbH in Berlin, Germany. Analysis briefly consisted of PCR amplification using universal forward primer U341F (5’‐CCTAYGGGRBGCASCAG‐‘3) and universal reverse primer U806R (5′‐GGACTACNNGGGTATCTAAT‐3′) targeting the V3‐V4 16S DNA region (Klindworth *et al.*, 2013). The protocol consisted of 10 cycles of touchdown PCR (annealing 61–55°C, decreasing 0.6°C per cycle), followed by 26 standard PCR cycles at an annealing temperature of 55°C. Quality control (using agarose gel electrophoresis), library preparation including tagging, equimolar mixing and clean‐up then was completed. 16S rRNA gene sequencing was performed on Illumina MiSeq V3 (2 × 300 bp).

Bioinformatics analysis consisted of inline barcode de‐multiplexing, adaptor clipping, and amplicon pre‐processing using Mothur (Schloss *et al.*, 2009), pair joining, filtering, alignment against Silva (128) 16S, subsampling 5000–25 000 reads per sample, denoising and chimera removal. OTU picking used Mothur clustering aligned sequences at 97% identity (see Figs S1–S6 for details). Additional OTU analysis was undertaken to confirm and complement the work of LGC Genomics, including assignment of taxonomy on the Greengenes database (version 13_8). Predominant OTUs were defined as having ≥ 0.5% relative abundance in any sample as by Narihiro *et al.* (2015) and Kuroda *et al. *(2016). In this case, these predominant OTUs equated to between 93% and 99% coverage in any sample. Phylogenetic analysis of predominant OTUs was performed with the ARB programme (version 6.0.4, Ludwig *et al. *(2004)), using neighbour‐joining and parsimony methods with 1000 bootstrap replication (McDonald *et al.*, 2012; Kuroda *et al.*, 2016).

### Statistical analysis

Alpha diversity and β‐diversity based on weighted UniFrac distances were calculated in QIIME. PRIMER 7 (PRIMER‐E, Plymouth, UK) was used for principal component analysis (PCA); metric‐multidimensional scaling (MDS); permutational analysis of variance (PERMANOVA) with 1000 permutations; analysis of similarities (ANOSIM); RELATE; BEST; and DistLM (distance‐based linear model) of the weighted UniFrac distances (even sampling at 12 069 reads) using Bray–Curtis after square root transformation, and physiochemical data (Ling *et al.*, 2016; Mei *et al.*, 2016b). Observed OTUs, Chao1 and Simpson’s Indices were plotted and compared using ANOVA (analysis of variance) with Tukey comparison in Minitab 17 (Leadtools Technologies Inc, Charlotte, NC, USA, version 17.1.0, 2014) with group significant differences compared in STAMP v2.1.3 using the *t*‐test. Significance was defined as 95% confidence in differences (i.e. *P* < 0.05). These sequence data have been submitted to the NCBI GenBank under Accession number MG808422‐MG811525.

## Conflict of interest

None declared.

## Supporting information


**Fig. S1.** Time‐course performance data for reactor operations post‐acclimation, i.e., (A) pH, (B) VS and (C) Total VFA, and for volumetric methane yields for each feeding frequency, designated (D) 5/7, (E) 3/7, (F) 1/7, (G) 1/14 and (H) 1/21. Adapted from Zealand et al., (2017).
**Fig. S2.** Microbial composition at phylum level. Each section represents initial inoculum, FF (5/7, 3/7, 1/7, 1/14, and 1/21) across time with each split into OLR1 and OLR2.
**Fig. S3.** Phylogenetic tree of shared predominant OTUs (only ≥ i0.5% abundance).
**Fig. S4.** Predominant OTUs (≥ 0.5% abundance) grouped based on ARB phylogenetic tree construction for OLR1, OLR2‐T, OLR2‐S, and, OLR2‐F. Area of bubbles represents relative abundance. ‘Inoc’ = inoculum. Letters in brackets under “Taxonomy” equate to classification
**Fig. S5.** Extended error bar plot showing predominant OTUs that have significantly different abundances between organic loading conditions, (A) OLR1 and OLR2‐T, (B) OLR2‐T and OLR2‐S, and, (C) OLR2‐S and OLR2‐F. Only OTUs with ≥ 0.5% abundance are shown and bold type indicates OTUs that appear in more than one panel.
**Fig. S6.** Shared predominant OTUs (only ≥ 0.5% abundance) based on sample appearances; i.e. in OLR1, OLR2‐T, OLR2‐S, and/or OLR2‐F. Area of bubbles represents relative abundance and in the case of shared OTUs, closeness to an OLR ‘hub’ indicates higher abundance; e.g. OTU 001 is nearer to OLR2‐S than OLR1. Bold indicates OTUs shared by two conditions and bold/underlined indicates OTUs shared by three conditions.
