# Supplementary material for: Molecular microbial ecology of stable versus failing rice straw anaerobic digesters
Source: Microb Biotechnol. 2019 Jun 10;12(5):879–91. doi: 10.1111/1751-7915.13438 (PMC6681398; doi:10.1111/1751-7915.13438)

**Supporting Information**

**Molecular Microbial Ecology of Stable versus Failing Rice Straw Anaerobic Digesters**

A. M. Zealand^a^, R. Mei^b^, A. P. Roskilly^c^, W. T. Liu^b^ and D. W. Graham^a*^

^a^ School of Engineering, Newcastle University, Newcastle upon Tyne, NE1 7RU, UK

^b^ Department of Civil and Environmental Engineering, University of Illinois at Urbana-Champaign, 205 North Mathews Ave, Urbana, IL 61801, USA

^c^ Sir Joseph Swan Centre for Energy Research, Newcastle University, Newcastle upon Tyne, NE1 7RU, UK

^*^Corresponding author: Prof David W. Graham

School of Civil Engineering & Geosciences

Cassie Building

Newcastle University

Newcastle upon Tyne

United Kingdom NE1 7RU

Phone: +44-191-208-7930

E-mail: [david.graham@newcastle.ac.uk](mailto:david.graham@newcastle.ac.uk)

**Additional Information on Next Generation Sequencing (NGS):**

Underpinning PCR reactions used 1-10 ng of DNA extract (total volume = 1.0 μl), 15 pmol of each forward primer and reverse primer (in 20 μL volume of 1.0 x MyTaq buffer containing 1.5 units MyTaq DNA polymerase (Bioline) and 2.0 μl of BioStabII PCR Enhancer (Sigma). For each sample, forward and reverse primers had the same 10-nt barcode sequence. PCR always was carried out for 30 cycles using the following conditions: 2 min at 96°C for pre-denaturation; 96°C for 15 s, 50°C for 30 s, and 70°C for 90 s.

Rough DNA concentrations of amplicons was determined using gel electrophoresis. About 20 ng amplicon DNA of each sample were pooled for up to 48 samples carrying different barcodes. If PCRs showed low yields, samples were amplified for an additional 5 cycles. Amplicon pools were purified with one volume AMPure XP beads (Agencourt) to remove primer dimer and other small mispriming products, followed by an additional purification on MinElute columns (Qiagen). About 100 ng of each purified amplicon pool DNA was used to construct Illumina libraries using the Ovation Rapid DR Multiplex System 1-96 (NuGEN). Illumina libraries were pooled and sizes selected by preparative gel electrophoresis. Sequencing was done on an Illumina MiSeq using V3 Chemistry (Illumina).

A total of 910,226 quality filtered samples were obtained from 39 samples with the number of 16S rDNA sequences ranging from 12,069 to 58,577 (mean, 23,339). After removing chimera sequences, operational taxonomic units (OTUs) were picked according to 97% similarity, which represented 9,720 OTUs. The alpha and beta diversity of the microbial community then were assessed.

**Supplementary Figures:**

**Fig. S1** Time-course performance data for reactor operations post-acclimation, i.e., **a)** pH, **b)** VS and **c)** Total VFA, and for volumetric methane yields for each feeding frequency, designated **d)** 5/7, **e)** 3/7, **f)** 1/7, **g)** 1/14 and **h)** 1/21. Adapted from Zealand *et al.*, (2017).

**Fig. S2** Microbial composition at phylum level. Each section represents initial inoculum, FF (5/7, 3/7, 1/7, 1/14, and 1/21) across time with each split into OLR1 and OLR2.

**Fig. S3** Phylogenetic tree of shared predominant OTUs (only ≥ 0.5 % abundance)

**Fig. S4** Predominant OTUs (≥ 0.5 % abundance) grouped based on ARB phylogenetic tree construction for OLR1, OLR2-T, OLR2-S, and, OLR2-F. Area of bubbles represents relative abundance. ‘Inoc’ = inoculum. Letters in brackets under “Taxonomy” equate to classification i.e., “O” = Order, “F” = Family, and “G” = Genus

**Fig. S5** Extended error bar plot showing predominant OTUs that have significantly different abundances between organic loading conditions, a) OLR1 and OLR2-T, b) OLR2-T and OLR2-S, and, c) OLR2-S and OLR2-F. Only OTUs with ≥ 0.5 % abundance are shown and bold type indicates OTUs that appear in more than one panel.

**Fig. S6** Shared predominant OTUs (only ≥ 0.5 % abundance) based on sample appearances; i.e. in OLR1, OLR2-T, OLR2-S, and/or OLR2-F. Area of bubbles represents relative abundance and in the case of shared OTUs, closeness to an OLR ‘hub’ indicates higher abundance; e.g. OTU 001 is nearer to OLR2-S than OLR1. Bold indicates OTUs shared by two conditions and bold/underlined indicates OTUs shared by three conditions.


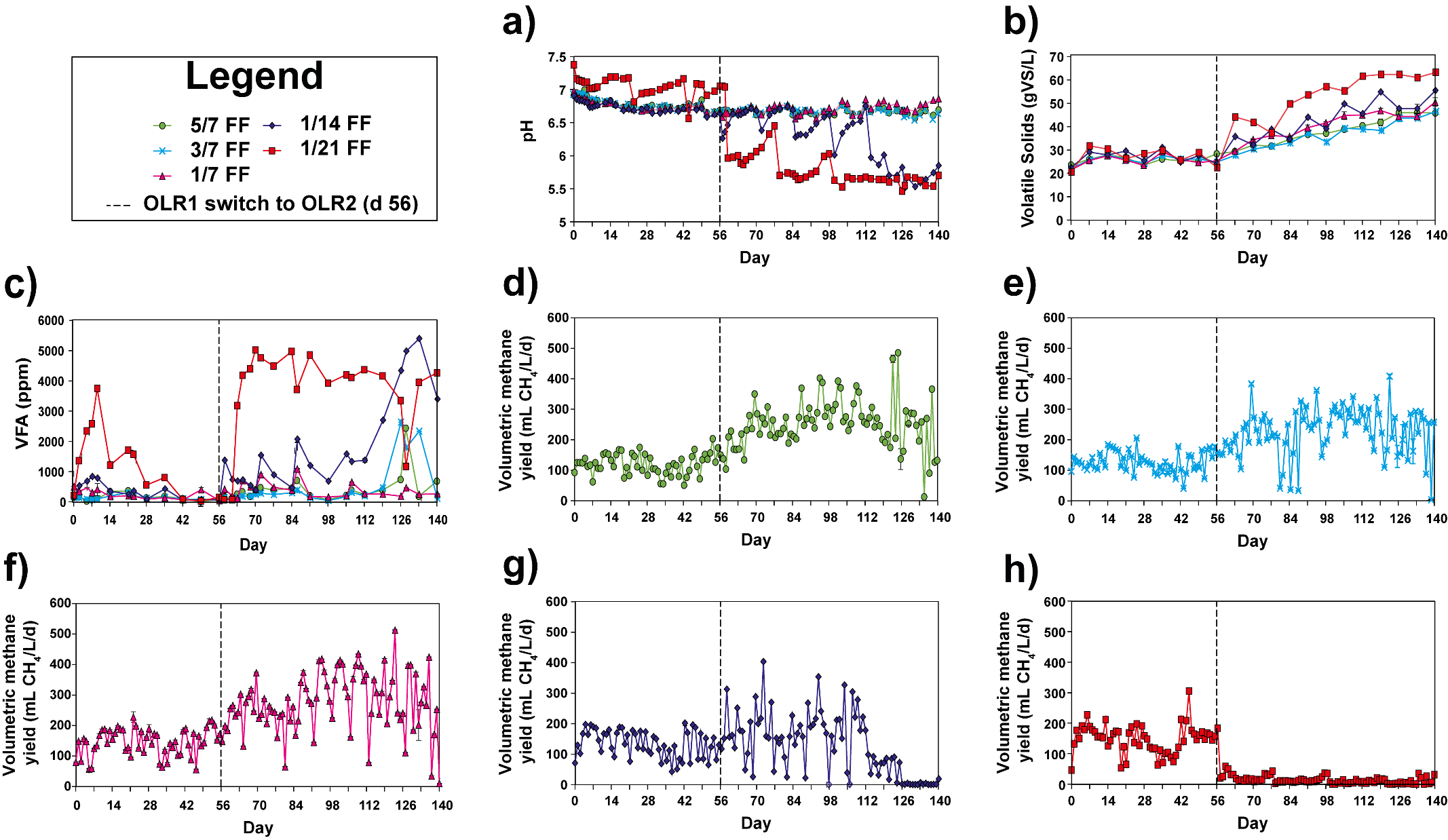


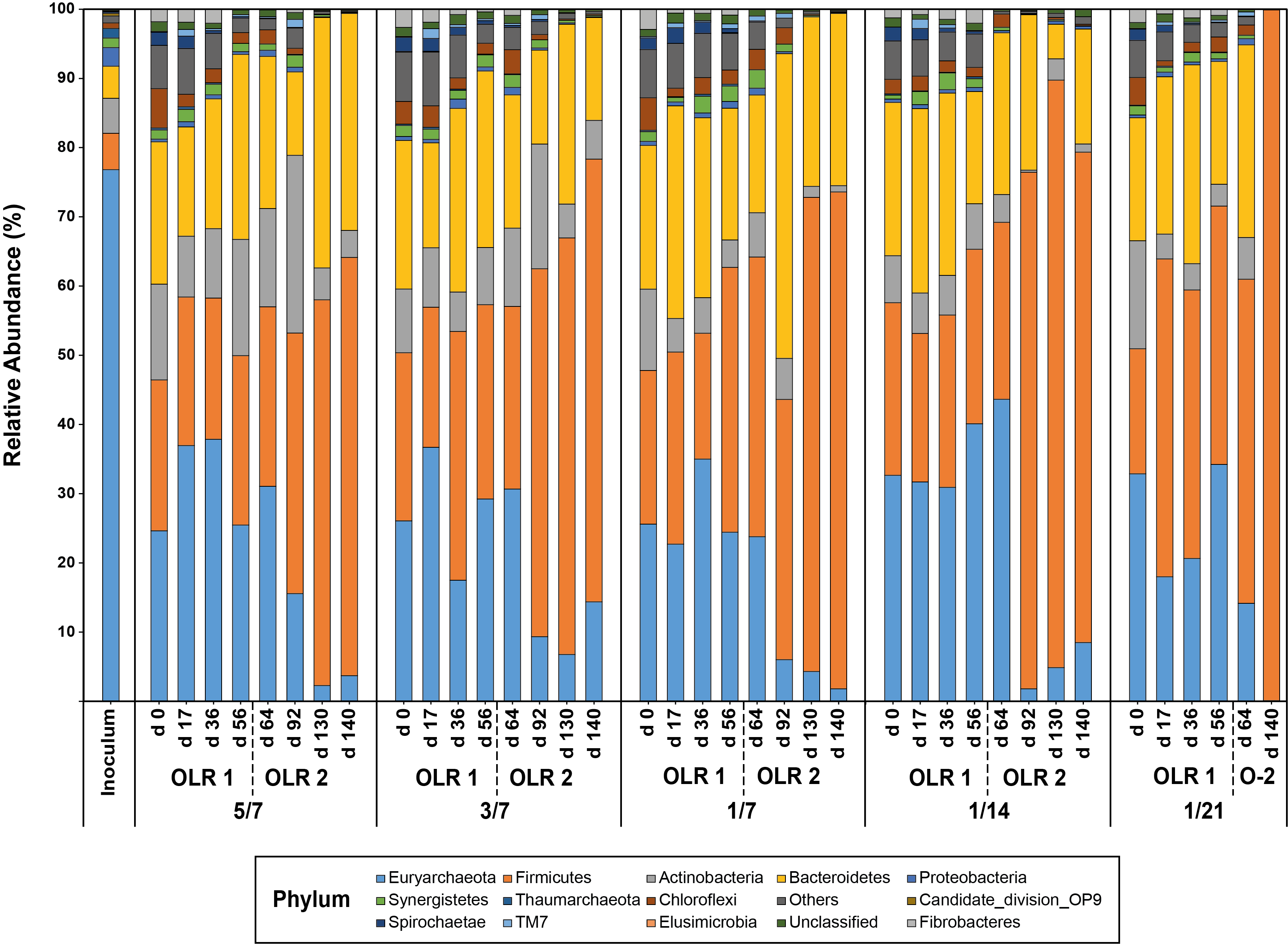


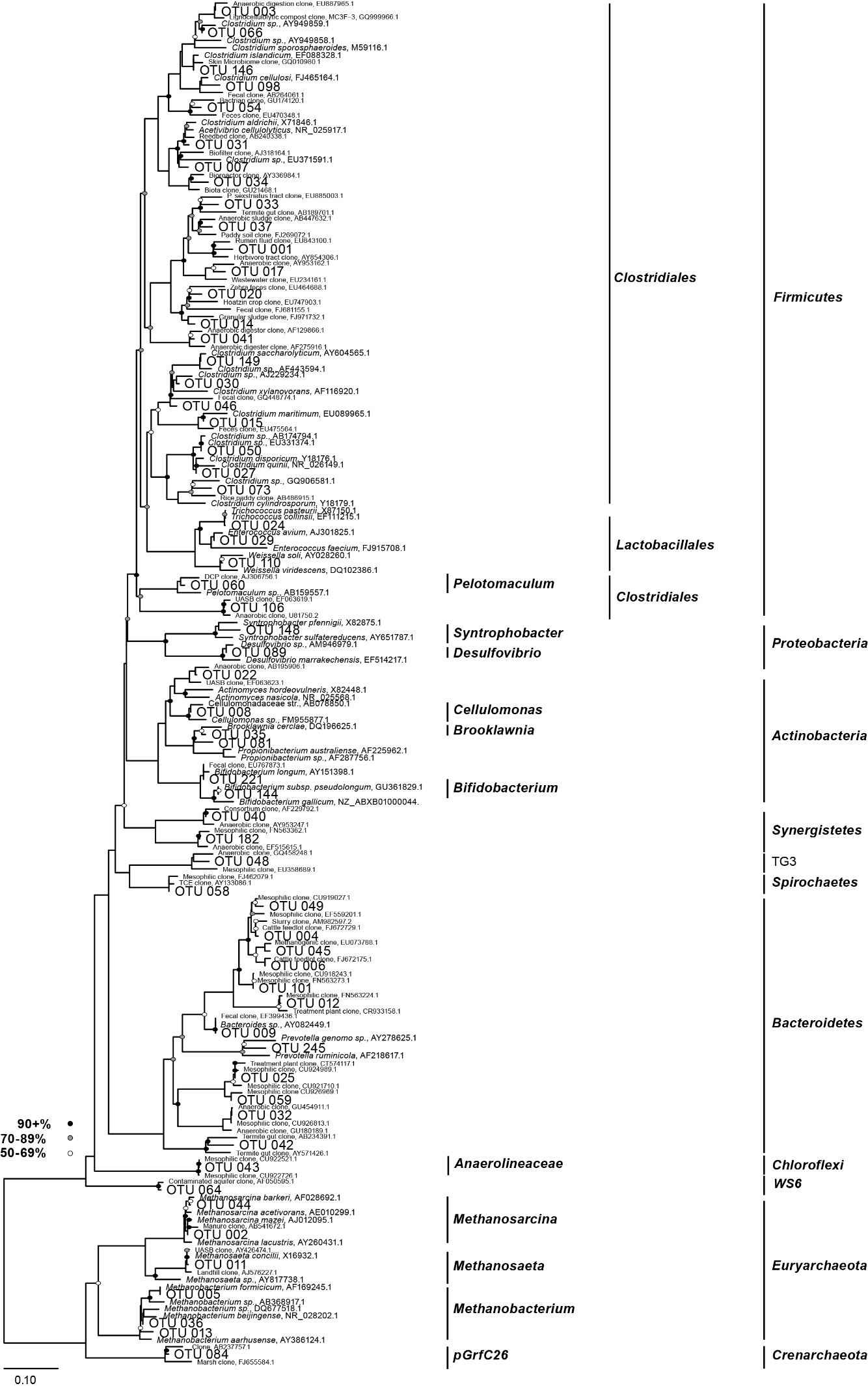


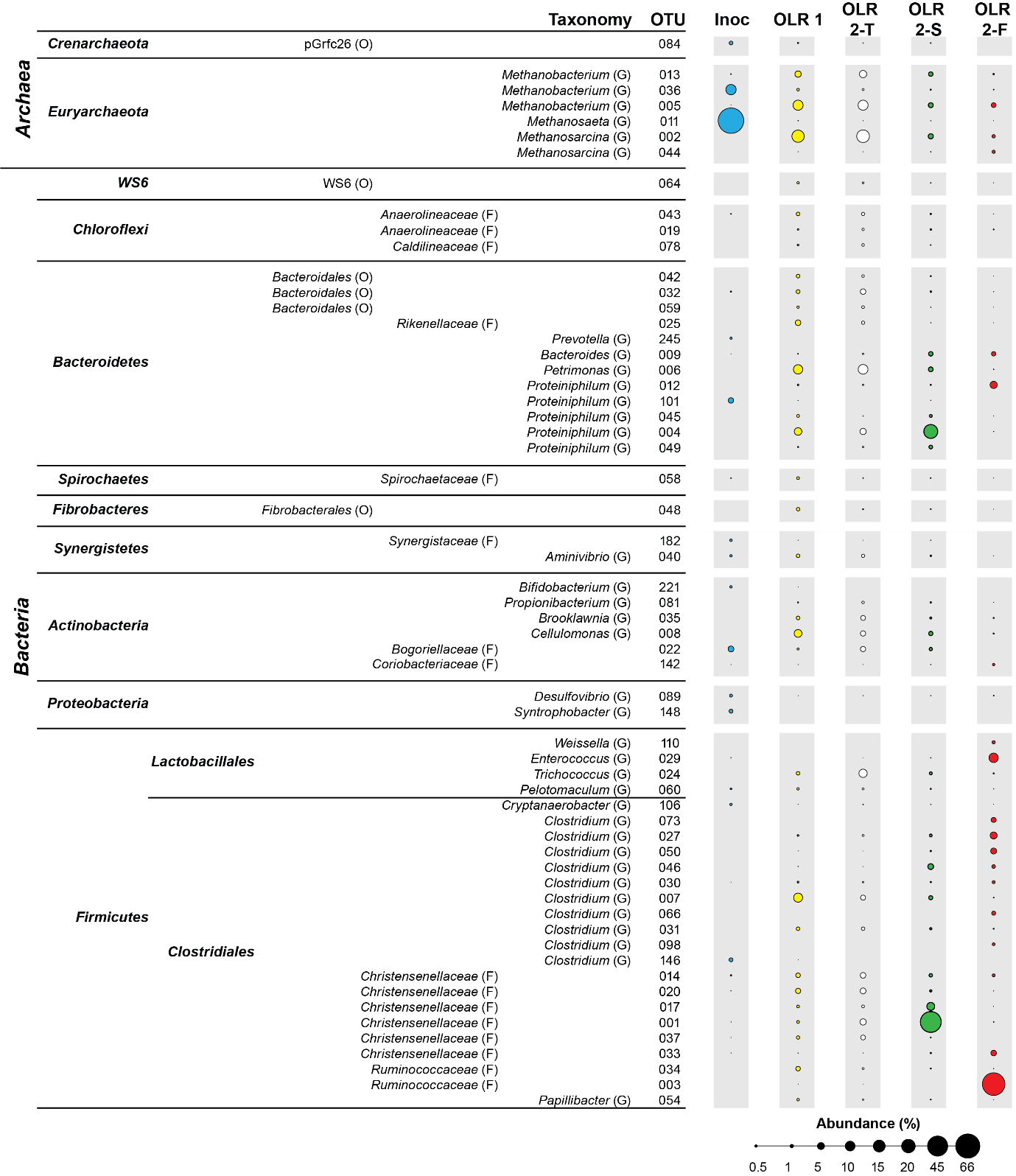


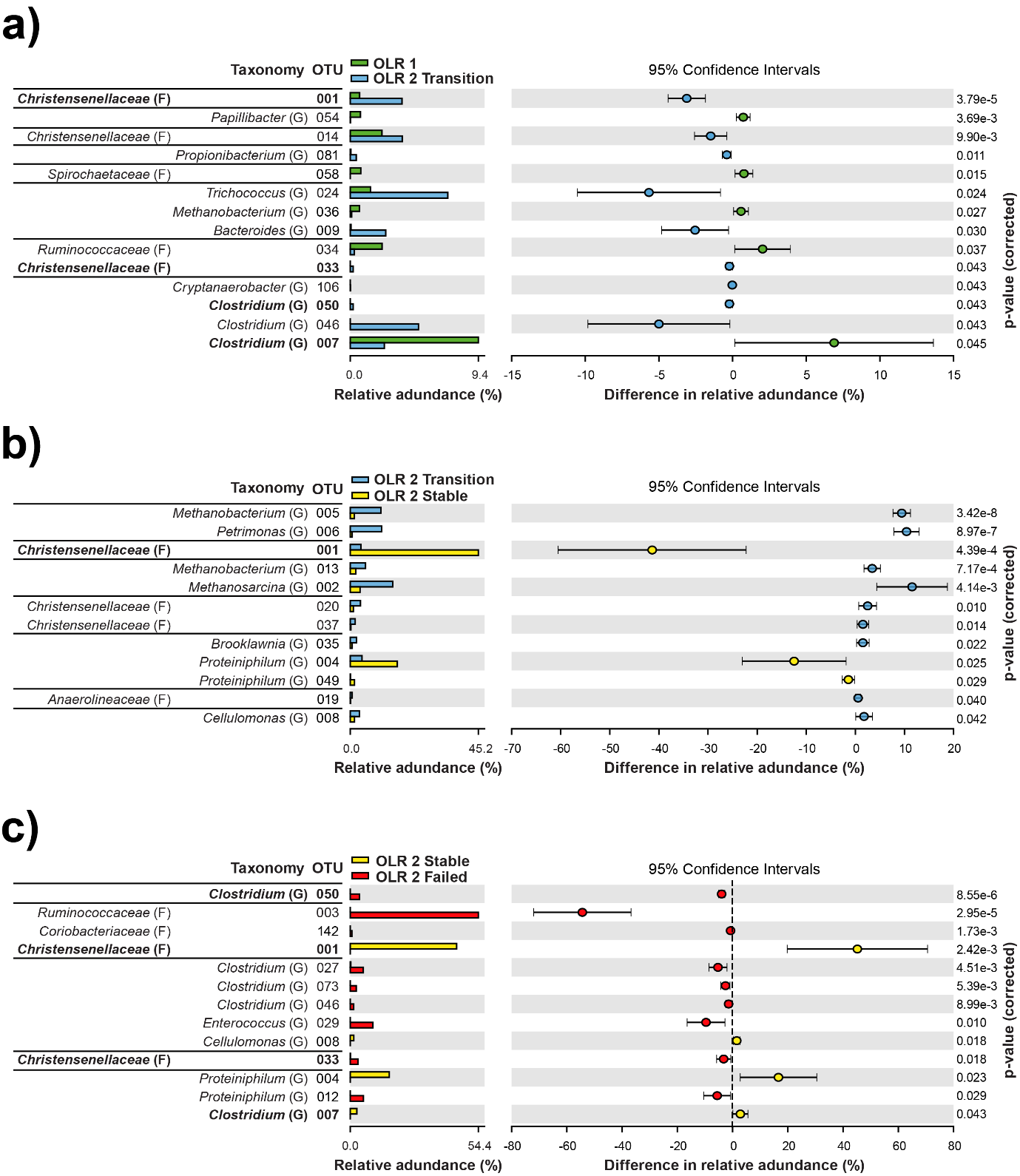


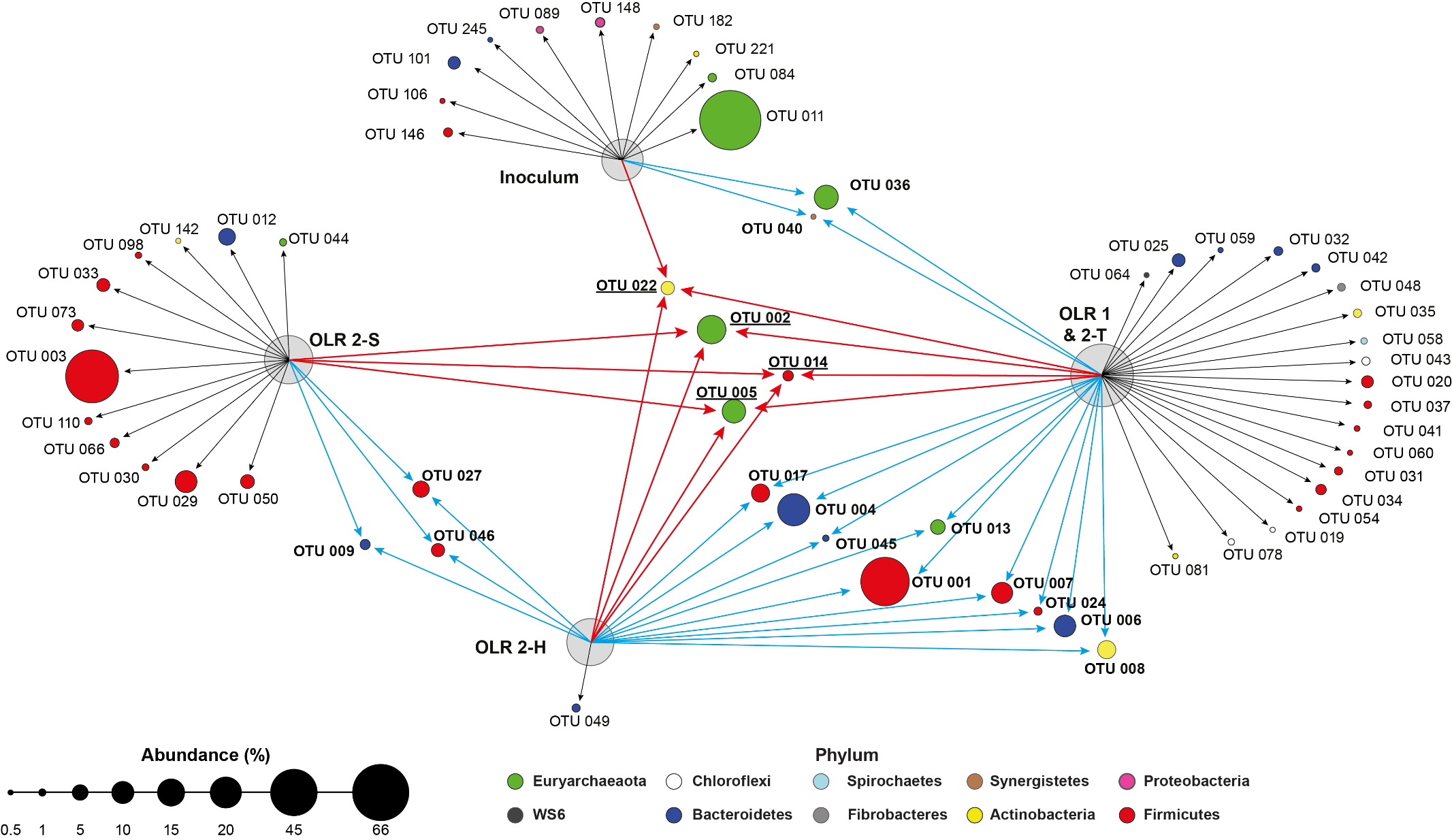

Supplement: Supplementary file 1 — Fig. S1. Time‐course performance data for reactor operations post‐acclimation, i.e., (A) pH, (B) VS and (C) Total VFA, and for volumetric methane yields for each feeding frequency, designated (D) 5/7, (E) 3/7, (F) 1/7, (G) 1/14 and (H) 1/21. Adapted from Zealand et al., (2017). Fig. S2. Microbial composition at phylum level. Each section represents initial inoculum, FF (5/7, 3/7, 1/7, 1/14, and 1/21) across time with each split into OLR1 and OLR2. Fig. S3. Phylogenetic tree of shared predominant OTUs (only ≥ i0.5% abundance). Fig. S4. Predominant OTUs (≥ 0.5% abundance) grouped based on ARB phylogenetic tree construction for OLR1, OLR2‐T, OLR2‐S, and, OLR2‐F. Area of bubbles represents relative abundance. ‘Inoc’ = inoculum. Letters in brackets under “Taxonomy” equate to classification Fig. S5. Extended error bar plot showing predominant OTUs that have significantly different abundances between organic loading conditions, (A) OLR1 and OLR2‐T, (B) OLR2‐T and OLR2‐S, and, (C) OLR2‐S and OLR2‐F. Only OTUs with ≥ 0.5% abundance are shown and bold type indicates OTUs that appear in more than one panel. Fig. S6. Shared predominant OTUs (only ≥ 0.5% abundance) based on sample appearances; i.e. in OLR1, OLR2‐T, OLR2‐S, and/or OLR2‐F. Area of bubbles represents relative abundance and in the case of shared OTUs, closeness to an OLR ‘hub’ indicates higher abundance; e.g. OTU 001 is nearer to OLR2‐S than OLR1. Bold indicates OTUs shared by two conditions and bold/underlined indicates OTUs shared by three conditions. [file MBT2-12-879-s001.docx]
